# Unravelling the Intricate Link: Mast Cells and Estrogen-Induced Pain Sensitization in Endometriosis

**DOI:** 10.7150/ijbs.116635

**Published:** 2025-09-21

**Authors:** Jianzhang Wang, Xinqi Mao, Libo Zhu, Xinmei Zhang

**Affiliations:** 1 Department of Gynecology, Women's Hospital, School of Medicine, Zhejiang University, Hangzhou, Zhejiang, P. R. China, 310006.; 2 Zhejiang Provincial Key Laboratory of Precision Diagnosis and Therapy for Major Gynecological Diseases, Women's Hospital, Zhejiang University School of Medicine, Hangzhou, Zhejiang, P. R. China, 310006.

**Keywords:** endometriosis, pain, mast cells, estrogen, nerve fibres

## Abstract

Endometriosis is a prevalent gynaecological disorder characterized by estrogen-dependent lesions. Pain—particularly dysmenorrhea, chronic pelvic pain, and dyspareunia—is the hallmark symptom of endometriosis. While pain mechanisms remain poorly understood, mast cells are now recognized as central mediators of estrogen-induced pain sensitization. Estrogen drives lesion growth and directly activates mast cells within lesions. Upon activation, mast cells release specific mediators, such as histamine and fibroblast growth factor 2 (FGF2), which enhance peripheral pain signalling and drive central sensitization through elevated responsiveness of dorsal horn neurons and increased neurotransmitter release in the spinal dorsal horn. This complex neuroimmune interaction between mast cells and nerve fibres in endometriotic lesions forms a positive feedback loop that amplifies pain. Targeting mast cells and their specific mediators represents a novel therapeutic strategy for pain management, particularly in cases of refractory pain. Further research into mast cell-mediated mechanisms will enable personalized and targeted therapies, revolutionize care and improve the quality of life for patients with endometriosis.

## 1. Introduction

### 1.1 Background of Endometriosis

#### 1.1.1 Definition and Prevalence

Endometriosis is a prevalent and complex gynaecological disorder that significantly affects the lives of many women worldwide. Endometriosis is defined as a condition in which tissue similar to the endometrium, the lining of the uterus, grows outside the uterus. This abnormal growth can occur in various locations, most commonly in the pelvic cavity, including on the ovaries, fallopian tubes, and pelvic peritoneum. In rare cases, lesions can also be found in distant sites such as the lungs, brain, or extremities^1^,^2^.

The global prevalence of endometriosis is estimated to be approximately 10% of women of reproductive age, which translates to approximately 190 million women worldwide. The epidemiology of endometriosis significantly varies in terms of prevalence estimates and is influenced by study design, diagnostic methods, and data sources^1^,^3^. Health insurance data often underestimate the prevalence, with rates ranging from 0.19% to 2.0%, while clinical data, including cohort and systematic studies, report a higher prevalence of 6.82%. Population-based surveys and self-reported studies have shown a pooled prevalence of 6.56%, with some reports indicating a prevalence of up to 18.3%. Symptomatic patients, particularly those with chronic pelvic pain or infertility, exhibit much higher prevalence rates, with studies reporting rates of up to 44%^4^.

This disease has a profound impact on women's health and quality of life. Endometriosis is associated with a wide range of symptoms, the most prominent of which is pain. The pain experienced by patients can be severe and debilitating, leading to limitations in daily activities, work absenteeism, and reduced productivity. Moreover, endometriosis-related pain can also have a significant psychological impact, contributing to the development of depression, anxiety, and other mental health disorders. In addition to causing pain, endometriosis is a leading factor in infertility and affects many women who wish to conceive, thereby significantly affecting their reproductive health and family planning^5^,^6^.

#### 1.1.2 The Centrality of Pain in Endometriosis

**Figure 1:**
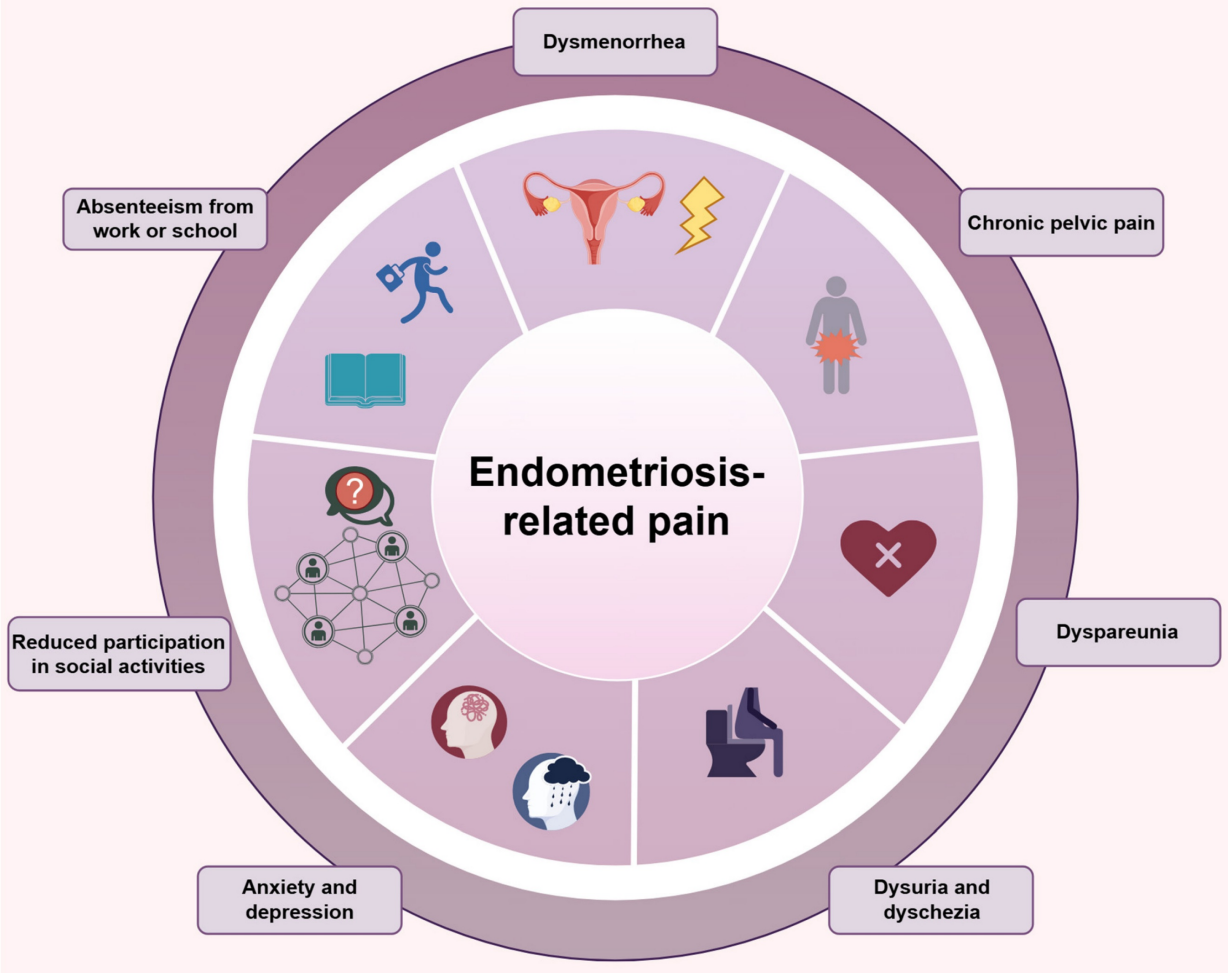
** Endometriosis-related pain.** The types of pain related to endometriosis include dysmenorrhea, chronic pelvic pain, dyspareunia, dysuria and dyschezia. These pain symptoms not only directly negatively affect the physical and mental health of patients, but also significantly affect their social and professional lives. (Created by Figdraw).

Pain is the most characteristic and distressing symptom of endometriosis, presenting in several forms that profoundly impact on patients' daily lives. Endometriosis-associated pain includes dysmenorrhea, chronic pelvic pain, dyspareunia, dysuria, and dyschezia (Figure 1). Dysmenorrhea, or painful menstruation, is among the most common symptoms of endometriosis. It is typically described as severe, cramping pain in the lower abdomen that occurs during the menstrual period. In endometriosis patients, dysmenorrhea is often secondary (developing after a period of normal menstruation) and progressive, indicating that the pain intensity and duration tend to increase over time. The pain can be so severe that it may interfere with normal daily activities, such as work, school, and social interactions. Many women with endometriosis-related dysmenorrhea may require bed rest during their menses and rely on pain medications, which may not always provide adequate relief^7^,^8^.

Chronic pelvic pain is another common manifestation of endometriosis. This is a persistent pain in the pelvic region that can occur throughout the menstrual cycle, not just during menstruation. Chronic pelvic pain can be a constant, dull ache or intermittent sharp pain. It can significantly affect a woman's quality of life, causing difficulties in performing routine physical activities, such as exercising, walking long distances, or even sitting for extended periods. It can also disrupt sleep patterns, leading to fatigue and further exacerbating the overall impact on the patient's well-being^9^,^10^.

Dyspareunia, or painful sexual intercourse, is also frequently associated with endometriosis. Pain can occur during penetration, deep thrusting, or even after sexual activity. This symptom can have a profound impact on a woman's sexual health and intimate relationships. Dyspareunia may lead to a decrease in sexual desire, avoidance of sexual activity, and emotional distress for both the patient and her partner. The psychological consequences of dyspareunia can be long-lasting and may contribute to relationship problems and negatively impact self-esteem^11^. These pain symptoms not only directly affect the physical and mental health of patients but also significantly affect their social and professional lives. Pain can lead to absenteeism from work or school, reduced participation in social activities, and increased health care utilization, resulting in a substantial economic burden on both the individual and society^12^ (Figure 1).

Endometriosis is a significant health issue with a high prevalence, and pain is at the core of this disease, causing a wide-ranging impact on the lives of affected women. Understanding the mechanisms underlying endometriosis-related pain, such as the role of mast cells in estrogen-induced pain sensitization, is crucial for the development of more effective treatment strategies.

### 1.2 The Role of Estrogen in Endometriosis

#### 1.2.1 Estrogen Dependence of Endometriotic Lesions

Estrogen plays a pivotal role in the development and progression of endometriosis, particularly in relation to the growth and survival of endometriotic lesions. Endometriotic tissue, like the normal endometrium, expresses estrogen receptors (ERs), mainly ERα, ERβ and G protein-coupled estrogen receptor 30 (GPR30). These receptors are key mediators through which estrogen affects on endometriotic cells^13^.

Estrogen promotes lesion proliferation, survival, and inflammatory responses via multiple pathways. Key mechanisms include local estrogen synthesis driven by aromatase overexpression, enabling the conversion of androgens to estrogens within lesions^14^. The predominance of estrogen receptors in ectopic tissues exacerbates progesterone resistance and upregulates the expression of proinflammatory cytokines. Therapeutic strategies targeting estrogen pathways—such as aromatase inhibitors and selective ERβ modulators—have demonstrated efficacy in preclinical and clinical trials, although long-term management remains limited by systemic estrogen suppression^15^,^16^.

#### 1.2.2 Estrogen-Induced Pain Sensitization

In endometriosis, estrogen-induced pain sensitization is a complex phenomenon that involves interactions among estrogen, the nervous system, and the immune system. Similar to normal endometrial tissue, ectopic endometrial tissue in patients with endometriosis undergoes cyclic changes in response to hormonal fluctuations. During the menstrual cycle, when estrogen levels are high, endometriotic lesions can release various inflammatory mediators and neurotransmitters, which can sensitize the surrounding nerves and lead to an enhanced perception of pain^17^.

One of the key mechanisms underlying estrogen-induced pain sensitization is the activation of nociceptors, the sensory nerve endings that detect pain. Estrogen can directly act on nociceptors, which may express estrogen receptors. By binding to these receptors, estrogen can modulate the activity of ion channels on nociceptors, such as transient receptor potential vanilloid 1 (TRPV1) channels. TRPV1 channels are involved in the detection of noxious heat and chemical stimuli. Studies have shown that estrogen can increase the expression and sensitivity of TRPV1 channels in nociceptors, increasing their responsiveness to pain-inducing stimuli^18^.

Estrogen also influences the release of neurotransmitters and neuropeptides in the pain signalling pathway. For example, it can increase the release of substance P, a neuropeptide that plays a crucial role in the transmission of pain signals. Substance P is released from the terminals of nociceptive neurons and acts on nearby cells, including immune cells and other neurons, to amplify pain signals. In endometriosis, elevated levels of estrogen-induced substance P release can lead to increased activation of second-order neurons in the spinal cord, resulting in central sensitization, a process in which the central nervous system becomes more sensitive to pain stimuli^19^.

Furthermore, estrogen can modulate the immune response in endometriotic lesions, and this immune‒endocrine interaction also contributes to pain sensitization. Hormonal fluctuations during the menstrual cycle, particularly increase in estrogen during the proliferative phase, stimulate endometrial cell proliferation, angiogenesis, and immune responses. This hormonal regulation is disrupted in endometriosis, leading to altered immune responses, with immune cells playing a key role in disease progression. Notably, mast cells have emerged as key effectors in estrogen-driven neuroimmune crosstalk. Estrogen interacts with both the innate and adaptive immune systems, influencing the immune environment in endometriotic lesions and contributing to the survival and invasion of ectopic endometrial tissue. The immune response in endometriosis is characterized by proinflammatory and immunosuppressive shifts that promote lesion persistence and immune evasion. These immune cells, which lead to the production of proinflammatory cytokines, sensitize nociceptors and enhance the pain signalling pathway^20^.

#### 1.2.3 Mechanisms of Pain Sensitization in Endometriosis

Pain processing involves complex neuroplastic changes, broadly categorized as peripheral and central sensitization. Peripheral sensitization denotes an enhanced responsiveness and reduced activation threshold of nociceptors—specialized sensory neurons in the dorsal root or trigeminal ganglia—within peripheral tissues. This state arises from local tissue injury, inflammation, or disease, leading to phenomena such as allodynia (pain from nonpainful stimuli) and hyperalgesia (heightened pain from painful stimuli). In endometriosis, peripheral sensitization manifests through distinct pathological alterations. A key feature is increased nerve fibre density within endometriotic lesions (peritoneal, ovarian, deep infiltrating), which significantly exceeds the levels found in normal tissue and is correlated with pain severity^21^. This increased fibre density is particularly notable for fibres expressing calcitonin gene-related peptide (CGRP)^22^. Furthermore, the expression of nociceptive receptors such as transient receptor potential vanilloid 1 (TRPV1) is upregulated^23^, a change directly linked to the intensity of dysmenorrhea. Concurrently, the peritoneal fluid develops a pro-sensitizing milieu, enriched with neurotrophins (such as NGF and BDNF) and growth factors (such as VEGF)^24^-^26^, which actively promote neuroangiogenesis and neurite outgrowth. Another significant mechanism is the perineural invasion (PNI) of endometriotic tissue along nerves, a process strongly associated with higher pain scores, dyspareunia, and sciatica^23^,^27^.

Conversely, central sensitization reflects an amplification of neural signalling within the central nervous system (CNS), fundamentally characterized by hyperexcitability of spinal cord dorsal horn neurons and altered processing in supraspinal brain regions. Key underlying mechanisms include synaptic facilitation, impaired descending inhibitory control, and structural and functional reorganization within the brain. Patients with endometriosis who experience persistent pelvic pain exhibit clear hallmarks of this central sensitization. Spinal cord hyperexcitability is a primary manifestation, as evidenced by widespread mechanical hyperalgesia, strongly suggesting the enhanced responsiveness of dorsal horn neurons^28^. Concurrently, alterations in brain structure and function are observed, including reduced grey matter volume in critical pain-processing regions such as the thalamus and insula, alongside increased resting-state functional connectivity within established pain networks^29^. Another key feature is dysregulation of the hypothalamic‒pituitary‒adrenal (HPA) axis, which manifests as blunted cortisol responses and impaired coping with stress^30^. Finally, enhanced central pain processing is demonstrable through functional neuroimaging studies, which consistently show heightened activation in pain-related brain regions when patients are exposed to both pelvic and peripheral noxious stimuli^31^.

Peripheral sensitization causes localized pain hypersensitivity directly related to a lesion (such as severe dysmenorrhea, dyspareunia, and localized hyperalgesia). In contrast, central sensitization drives more diffuse and widespread pain phenomena. This includes secondary hyperalgesia in areas distant from the lesion, persistent pain even after lesion removal^32^, and conditions such as widespread chronic pelvic pain and comorbid pain syndromes (such as migraines and bladder pain syndrome)^22^,^33^. These phenomena indicate a maladaptive state of the CNS. Crucially, the persistence of central sensitization is key to the transition to chronic, treatment-refractory pain.

## 2. Interaction between Estrogen and Mast Cells

### 2.1 Introduction to Mast Cells

Mast cells are immune cells that play crucial roles in immune and inflammatory responses. Mast cells originate from haematopoietic stem cells in the bone marrow. During their development, these precursor cells leave the bone marrow and circulate in the bloodstream as immature mast cells. Eventually, the immature cells migrate to various tissues throughout the body, where they complete their maturation process. Mast cells are widely distributed in the body, with a particular preference for tissues that are in contact with the external environment, such as the skin, respiratory tract, and gastrointestinal tract^34^.

One of the most well-known immune-related functions of mast cells is their role in allergic reactions. When a person is exposed to an allergen for the first time, the immune system produces immunoglobulin E (IgE) antibodies specific to that allergen. These IgE antibodies then bind to high-affinity IgE receptors on the surface of mast cells. Upon subsequent exposure to the same allergen, the allergen cross-links the IgE antibodies on the mast cell surface, triggering a series of intracellular signalling events. This leads to the rapid release of preformed mediators stored in mast cell granules, such as histamine, heparin, and proteases. Histamine, for example, causes vasodilation, increased vascular permeability, smooth muscle contraction, and itching, which are characteristic symptoms of allergic reactions. In addition to the immediate-phase response mediated by preformed mediators, mast cells also produce and secrete newly synthesized lipid mediators and cytokines during a later phase of the response. These late-phase mediators further amplify the inflammatory response, recruit other immune cells to the site of inflammation, and contribute to the development of chronic allergic inflammation^35^.

Mast cells also play a role in the defence against pathogens. These cells can recognize pathogens through pattern-recognition receptors, such as Toll-like receptors (TLRs), which are expressed on their surface. Activation of TLRs on mast cells by pathogen-associated molecular patterns leads to the release of proinflammatory mediators and cytokines. These mediators can directly act against pathogens, for example, by increasing the permeability of blood vessels to allow the entry of immune cells and antimicrobial substances to the site of infection. Mast cells can also interact with other immune cells, such as macrophages and T lymphocytes, to coordinate the immune response against pathogens. For instance, mast cells can secrete cytokines that activate macrophages, enhancing their phagocytic and microbicidal abilities^36^,^37^.

### 2.2 Effects of Estrogen on Mast Cell Behaviour

#### 2.2.1 Mast Cell Accumulation and Activation in Endometriotic Lesions

Studies have demonstrated the accumulation of mast cells in endometriotic lesions^38^-^40^. In patients with endometriosis, widespread infiltration of a large number of mast cells was noted throughout the stromal lesions. The number of degranulated mast cells in the stroma of lesion samples from endometriosis patients was significantly greater than that in samples from eutopic endometrium or the laparoscopically retrieved normal uterine serosa^41^. Inflammatory signals (such as IL-6 and TNF-α) upregulate aromatase expression, increasing local estrogen biosynthesis in endometriotic lesions^42^. This ectopic estrogen synthesis potently amplifies mast cell activation through direct binding to estrogen receptors and GPR30 on mast cells, triggering degranulation^24^,^43^. Concurrently, estrogen-stimulated mast cells release proinflammatory mediators, which further induce aromatase activity. This reciprocal interaction establishes a self-reinforcing estrogen-inflammatory feedforward loop, driving sustained mast cell activation and propagating a vicious cycle. The promotion of mast cell proliferation and survival by estrogen can lead to an increased population of activated mast cells in the endometriotic microenvironment^44^. The activation status of mast cells in endometriotic lesions has also been studied. Activation of mast cells can be detected by the expression of various markers, such as the release of granule-associated mediators (e.g., histamine and tryptase), upregulation of activation-related genes, and changes in cell morphology. In endometriotic lesions, mast cells are activated, as indicated by the increased expression of tryptase, a specific marker of mast cell activation^24^. These mast cells can then release a variety of inflammatory mediators, which play key roles in the development of endometriosis-related inflammation and pain. Understanding these mechanisms is crucial for developing targeted therapies to disrupt the estrogen-mast cell axis in endometriosis.

#### 2.2.2 Mediator Release from Mast Cells

Estrogen has a significant effect on the release of mast cell mediators, which are key factors in the development of pain sensitization in endometriosis. One of the most well-studied mediators is histamine, which is stored in mast cell granules and is rapidly released upon mast cell activation. This increased histamine release can^42^,^43^ have multiple effects on pain sensitization. Histamine acts on histamine receptors, such as H1 and H2 receptors, on nerve endings. Activation of H1 receptors on nociceptors can lead to the depolarization of nerve fibres, increasing their excitability and the transmission of pain signals. In addition, cytokines and chemokines are other mediators released by mast cells, and estrogen can modulate their production and release. In endometriosis, estrogen upregulates the expression and release of many cytokines and chemokines in mast cells. These cytokines can act on various cell types in the endometriotic microenvironment, including immune cells and nerve cells^44^,^45^. In summary, estrogen-induced changes in the release of mast cell mediators, including histamine, cytokines and chemokines, can have a profound effect on pain sensitization in patients with endometriosis. These mediators can act on nerve endings, immune cells, and the extracellular matrix, leading to the activation and sensitization of pain signalling pathway and the development of chronic pain in endometriosis patients.

### 2.3 Signalling Pathways Triggered by Estrogen in Mast Cells in Endometriosis

Estrogen receptors, including ERα, ERβ and GPR30, have been detected in mast cells, but their distribution and relative expression levels can vary depending on the tissue source and the activation state of the mast cells. When estrogen binds to its receptors on mast cells, it initiates a series of intracellular signalling cascades that can significantly affect mast cell function.

The classical genomic pathway involves the binding of estrogen to ERs, which are located in the cytoplasm in an inactive state bound to heat shock proteins. Upon estrogen binding, the heat shock proteins dissociate, and the estrogen-ER complex is translocated to the nucleus. In the nucleus, the complex binds to specific estrogen-response elements (EREs) in the promoter regions of target genes. This binding results in the recruitment of coactivators and other transcriptional machinery, leading to the regulation of gene expression. Our previous study revealed that elevated estrogen levels in ovarian ectopic lesions can activate the inflammasome pathway of NOD-like receptor family pyrin domain-containing 3 by enhancing transcription via ER-α through ERE in mast cells. This leads to increased NLRP3 expression and potassium efflux, which triggers the recruitment of inflammasomes, the activation of caspase-1, and the production and secretion of active IL-1β, potentially driving the development of endometriosis^46^.

In addition to the classical genomic pathway, a nongenomic pathway can also be activated rapidly upon estrogen binding to ERs on mast cells. This nongenomic pathway involves the activation of membrane-associated ERs, which can interact with various signalling molecules at the cell membrane. Using a combination of in vivo and in vitro models, we demonstrated that a high concentration of estrogen in the local endometriotic environment stimulated the secretion of fibroblast growth factor 2 (FGF2) in mast cells through GPR30 via the MEK/ERK pathway. Elevated FGF2 levels in mast cells within ovarian endometriotic lesions could contribute to the worsening of endometriosis-related pain^43^.

Overall, the signalling pathways triggered by estrogen-ER binding in mast cells are complex and can have both direct and indirect effects on mast cell function, which is crucial for understanding their role in estrogen-induced pain sensitization in endometriosis.

## 3. Mast Cell-Mediated Pain Sensitization and Underlying Molecular Mechanisms

### 3.1 Role of Inflammatory Mediators

#### 3.1.1 Histamine

Histamine is among the primary mediators released by mast cells and plays a crucial role in pain sensitization in endometriosis. When mast cells are activated, they rapidly release histamine from their granules into the extracellular environment. Histamine affects nerve endings through specific histamine receptors, primarily H1 and H2 receptors, which are expressed on nociceptive neurons^47^.

Activation of H1 receptors on nociceptive neurons leads to a series of intracellular events that ultimately result in pain and itching sensations. The binding of histamine to H1 receptors activates phospholipase C (PLC)-mediated signalling pathway. PLC cleaves phosphatidylinositol 4,5-bisphosphate (PIP2) into inositol trisphosphate (IP3) and diacylglycerol (DAG). IP3 binds to receptors on the endoplasmic reticulum, causing the release of calcium ions (Ca^2+^) from intracellular stores. An increase in the intracellular Ca^2+^ concentration leads to the depolarization of nociceptive neurons, making them more excitable. This increased excitability allows the neurons to fire action potentials more readily in response to pain-inducing stimuli, thereby increasing the transmission of pain signals to the central nervous system^48^.

In addition to its effects on neuronal excitability, histamine can directly stimulate nociceptive nerve endings, leading to the generation of pain signals. It can open certain ion channels, such as TRPV1 channels, on nociceptive neurons. TRPV1 channels are sensitive to noxious heat, acidity, and certain chemical stimuli. Activation of TRPV1 channels by histamine can increase the sensitivity of nociceptive neurons to these stimuli, contributing to the perception of pain^49^,^50^. Moreover, histamine-induced activation of nociceptive neurons can lead to the release of neurotransmitters, such as substance P and calcitonin gene-related peptide (CGRP), which are important for the transmission and amplification of pain signals. Substance P, for example, can act on nearby neurons and immune cells, further sensitizing the pain signalling pathway^47^.

The role of histamine in endometriosis-related pain is also supported by clinical and experimental evidence. In endometriosis patients, elevated levels of histamine have been detected in peripheral blood and endometriotic lesions^51^. Blocking histamine receptors with antihistamine drugs has been shown to reduce pain symptoms in some patients. In animal models of endometriosis, the administration of histamine can exacerbate pain-related behaviours, whereas the inhibition of histamine release or the blockade of histamine receptors can alleviate these behaviours, further highlighting the importance of histamine in mast cell-mediated pain sensitization in endometriosis^52^,^53^.

#### 3.1.2 Cytokines and Chemokines

Mast cells play a significant role in endometriosis through the secretion of cytokines and chemokines that contribute to inflammation, immune cell recruitment, and tissue remodelling in the pelvic environment. These factors play a critical role in the pathophysiology of endometriosis, leading to symptoms such as chronic pelvic pain and the growth of ectopic endometrial tissue. Interleukin-6 (IL-6) is a key cytokine released by mast cells in endometriosis. IL-6 can have multiple effects on pain signalling. It can directly act on nociceptive neurons to increase their excitability. IL-6 binds to its specific receptors on nociceptive neurons, triggering the activation of the Janus kinase (JAK)-signal transducer and activator of transcription (STAT) signalling pathway. This activation leads to the upregulation of genes encoding ion channels and neurotransmitter receptors on nociceptive neurons. For example, the expression of TRPV1 channels may increase, increasing the sensitivity of neurons to noxious stimuli. IL-6 can also promote the release of other proinflammatory mediators from immune cells, such as macrophages and T lymphocytes, further amplifying the inflammatory response and contributing to pain sensitization. Tumour necrosis factor-α (TNF-α) is another important cytokine released by mast cells. TNF-α can directly sensitize nociceptive neurons. It activates intracellular signalling pathways, such as the nuclear factor-κB (NF-κB) pathway, in nociceptive neurons. Activation of the NF-κB pathway leads to the production of proinflammatory mediators within the neurons themselves. These mediators can then act on ion channels, such as voltage-gated sodium channels, which are important for the generation and propagation of action potentials in nociceptive neurons. By modulating the function of these ion channels, TNF-α can increase the excitability of nociceptive neurons and enhance the transmission of pain signals^54^. Chemokines, such as CCL2 (monocyte chemoattractant protein-1, MCP-1) and CCL3 (macrophage inflammatory protein-1α, MIP-1α), are also released by mast cells in endometriosis. CCL2 and CCL3 play important roles in recruiting immune cells to the site of inflammation. These chemokines bind to specific chemokine receptors on immune cells, such as monocytes, macrophages, and T lymphocytes, promoting their migration towards the endometriotic lesions. The recruitment of these immune cells can lead to the further release of cytokines and other inflammatory mediators, creating a positive-feedback loop of inflammation. Additionally, chemokines can also directly act on nociceptive neurons. They can activate intracellular signalling pathways in nociceptive neurons, similar to those activated by cytokines, leading to an increase in their excitability and the release of neurotransmitters involved in pain transmission^45^.

Overall, the cytokines and chemokines released by mast cells in endometriosis contribute to the complex process of pain sensitization. They interact with each other, immune cells, and nociceptive neurons, leading to the activation and amplification of the pain-signalling pathway and ultimately resulting in the development of chronic pain in endometriosis patients.

### 3.2 Neuro-Immune Interactions

#### 3.2.1 Communication between Mast Cells and Nerve Fibres

In endometriotic lesions, mast cells and nerve fibres engage in complex physical and chemical interactions that play crucial roles in the development of pain. Physically, mast cells are often found in close proximity to nerve fibres, especially in the stromal areas of endometriotic implants. This close spatial arrangement allows for efficient chemical communication between the two cell types^55^.

The chemical interaction between mast cells and nerve fibres is mediated by the release of neurotransmitters and neuropeptides. One of the key neuropeptides involved in this interaction is substance P. Mast cells in endometriotic lesions can release histamine, which can stimulate the release of substance P from sensory nerve fibres. Substance P is a potent mediator of pain transmission. It is released from the terminals of nociceptive neurons, which are sensory neurons that detect noxious stimuli. Once released, substance P acts on nearby cells, including the mast cells themselves, through the neurokinin-1 receptor (NK-1R). Activation of NK-1Rs on mast cells can lead to further mast cell activation and the release of more inflammatory mediators, creating a positive feedback loop that amplifies the pain-signalling pathway^54^.

Calcitonin gene-related peptide (CGRP) is another important neuropeptide involved in the communication between mast cells and nerve fibres. CGRP is coreleased with substance P from sensory nerve fibres in response to noxious stimuli. In endometriotic lesions, CGRP can act on mast cells to modulate their function. For example, CGRP can increase the release of cytokines from mast cells, such as IL-6 and TNF-α. These cytokines can then act on nerve fibres to sensitize them, increasing their excitability and the transmission of pain signals. Conversely, mast cell-derived mediators can also regulate the release of CGRP from nerve fibres. For instance, histamine can stimulate the release of CGRP from sensory nerve endings, further contributing to the amplification of the pain response^45^.

This reciprocal activation establishes a self-sustaining positive feedback loop: mast cell-derived mediators sensitize nerves, whereas nerve-derived neuropeptides reactivate mast cells, continuously amplifying peripheral nociceptive signalling.

Notably, neuroimmune interactions vary significantly across endometriosis subtypes. Histological and transcriptomic analyses have shown that deep infiltrating endometriosis (DIE) lesions have features distinct from those of peritoneal or ovarian subtypes. These features include a higher density of mast cells, increased activation and degranulation of these cells, and a closer spatial proximity to nerve fibres (within 25 μm)^56^. This unique neuroinflammatory niche allows mast cell-derived mediators - such as histamine, tryptase, and nerve growth factor (NGF) - to directly modulate nociceptors. This phenomenon may drive the rich innervation seen in DIE lesions^57^,^58^ and is correlated with elevated clinical pain scores. In contrast, fewer mast cells and weaker neuroimmune interactions are present in peritoneal lesions. This finding aligns with the milder pain symptoms reported for this subtype^59^. Further spatial transcriptomics studies are warranted to characterize differential mast cell-nerve interactions among DIE, peritoneal, and ovarian endometriosis subtypes.

#### 3.2.2 Activation of the Central Nervous System

The activation of mast cells in endometriotic lesions can lead to activation of the central nervous system (CNS), resulting in central sensitization and enhanced pain perception.

Sustained nociceptive input from sensitized peripheral nerves (driven by mast cell mediators) increases neurotransmitter release in the spinal dorsal horn. Specifically, when mast cells in endometriotic lesions are activated, they release a variety of mediators, such as histamine, cytokines, and neuropeptides. These mediators can directly act on the terminals of primary afferent neurons, depolarizing them and generating action potentials. For example, histamine can open ion channels on the terminals of primary afferent neurons, leading to an influx of sodium ions and the depolarization of the neurons. Cytokines, such as IL-6 and TNF-α, can also sensitize the terminals of primary afferent neurons by modulating the function of ion channels and neurotransmitter receptors. Once activated, the primary afferent neurons transmit pain signals to the spinal cord.

In the spinal cord, primary afferent neurons synapse with second-order neurons in the dorsal horn. The release of neurotransmitters and neuropeptides from primary afferent neurons, such as substance P and glutamate, activates second-order neurons. The activation of these second-order neurons can lead to the release of more neurotransmitters and the activation of other neurons in the spinal cord, creating a complex network of neural activity. This process is known as wind-up, which is a form of central sensitization. Central sensitization results in increased responsiveness of the CNS to pain stimuli, leading to enhanced pain perception. Second-order neurons in the spinal cord can also send signals to higher-order brain regions, such as the thalamus, hypothalamus, and limbic system, which are involved in the perception, modulation, and emotional processing of pain. The activation of the central nervous system by mast cell-derived signals in endometriosis contributes to the complex pathophysiology of pain in this disease, involving both the sensory and emotional components of pain perception.

Additionally, mast cell-derived TNF-α/IL-6 promotes neuroinflammation by activating spinal microglia/astrocytes, which release additional pro-nociceptive mediators (such as BDNF and ATP)^45^.

Collectively, the mast cell-nerve feedback loop critically fuels central sensitization; this process amplifies central sensitization by expanding receptive fields (allodynia), prolonging poststimulus pain (temporal summation), and facilitating the transmission of ascending pain transmission to supraspinal sites (such as the thalamus and limbic system), thereby heightening pain perception and chronicity.

## 4. Therapeutic Implications

### 4.1 Current Treatments for Endometriosis-Related Pain

Pharmacological treatments for endometriosis-related pain include NSAIDs, hormonal therapies, and GnRH analogues. NSAIDs, such as ibuprofen, provide short-term pain relief by reducing inflammation but can cause gastrointestinal issues and kidney damage, and do not address the root cause of the disease. Hormonal therapies such as combined oral contraceptives and progestins suppress ovulation and menstruation, reducing endometriotic lesion growth, but may lead to side effects such as weight gain, mood changes, and headaches. GnRH analogues induce a temporary menopausal state by lowering estrogen levels, which alleviates pain, but prolonged use can cause hot flashes, vaginal dryness, and bone density loss. Although these pharmacological treatments can offer symptom relief, they do not provide a permanent solution and can cause significant side effects, which limits their long-term effectiveness in managing this condition^60^.

### 4.2 Mast-Cell-Targeted Therapies

#### 4.2.1 Antihistamines

Antihistamines function by blocking the effects of histamine, a key mediator released by mast cells. Histamine exerts its effects through binding to specific histamine receptors, primarily H1 and H2 receptors. In the context of endometriosis-related pain, antihistamines can potentially provide relief by interfering with the histamine-mediated signalling pathways that contribute to pain sensitization.

First-generation antihistamines, such as diphenhydramine and chlorpheniramine, have been used for many years. These drugs are nonselective in their activity and bind to both H1 and H2 receptors. In endometriosis, they may help to reduce pain by blocking H1 receptors on nociceptive neurons. However, the sedative effects limit their use, especially in patients who need to maintain normal daily activities. Second-generation antihistamines, such as loratadine, cetirizine, and fexofenadine, have been developed to address these limitations. In endometriosis, second-generation antihistamines can still effectively block the H1 receptor-mediated actions of histamine. They can prevent histamine-induced vasodilation, increased vascular permeability, and activate nociceptive neurons.

In addition to their direct effects on pain signalling pathways, antihistamines may also have anti-inflammatory properties. By blocking the actions of histamine, they can reduce the recruitment and activation of other immune cells in the endometriotic microenvironment. Histamine can attract eosinophils, neutrophils, and T lymphocytes to the site of inflammation, and by inhibiting this recruitment, antihistamines can help to dampen the overall inflammatory response associated with endometriosis. This anti-inflammatory effect can further contribute to the reduction in pain in endometriosis patients, as inflammation is a key factor in the development of pain sensitization^61^,^62^.

#### 4.2.2 Mast Cell Stabilizers

Mast cell stabilizers are a class of drugs that act by preventing mast cell degranulation, thereby inhibiting the release of inflammatory mediators, including histamine, cytokines, and proteases. These drugs work by targeting the intracellular signalling pathways involved in mast cell activation. One of the most well-known mast cell stabilizers is cromolyn sodium. Cromolyn sodium is thought to act by blocking calcium channels on mast cells. As calcium influx is a crucial step in mast cell activation, blocking these channels can prevent the activation of signalling pathways that lead to degranulation. In the context of endometriosis, mast cell stabilizers such as cromolyn sodium have the potential to be effective at reducing pain^63^. By preventing mast cell degranulation, they can reduce the levels of histamine and other inflammatory mediators in the endometriotic microenvironment. Reducing the release of these mediators can lead to a decrease in the activation and sensitization of nociceptive neurons, thereby alleviating pain. Another mast cell stabilizer, ketotifen, also has similar mechanisms of action^52^. In conclusion, the potential of mast cell stabilizers to target the mast cell-mediated pain pathway makes them attractive options for future therapeutic development.

In clinical trials, the antihistamine drug ebastine and the mast cell stabilizers ketotifen were found to reduce pain symptoms in patients with IBS and fibromyalgia 64-66. However, no randomized controlled trials (RCTs) or retrospective studies specifically testing antihistamines or mast cell stabilizers for endometriosis pain have been carried out currently. While antihistamines and stabilizers show preclinical efficacy, their clinical use in endometriosis remains experimental and warrants validation in human trials.

Notably, both antihistamines and mast cell stabilizers cause systemic side effects. First-generation antihistamines cause pronounced sedation and anticholinergic effects, whereas second-generation agents cause minimal central nervous system (CNS) toxicity because of their high peripheral H1 receptor selectivity and poor blood-brain barrier penetration^67^,^68^. Long-term antihistamine use may also contribute to metabolic issues such as obesity 69,70. Mast cell stabilizers commonly cause sedation, weight gain, dizziness, dry mouth, nausea, headache, and increased appetite^71^-^73^. Further research is needed to optimize their use, determine the most effective dosing regimens, prevent adverse reactions, and evaluate their long-term safety and efficacy in endometriosis patients.

Mast cells are mechanistically implicated in endometriosis-related pain and inflammation, highlighting their potential as therapeutic targets. Crucially, mRNA levels of the mast-cell-specific markers C-KIT and TPSAB1 (encoding tryptase) are significantly elevated in patients experiencing endometriosis pain compared with those without pain. The numbers of C-KIT-expressing mast cells and tryptase-expressing mast cells in ovarian endometriotic lesions are positively correlated with endometriosis pain. These elevated levels suggest the existence of a distinct “mast cell-rich” disease subtype. Therefore, mast cell markers, such as C-KIT and tryptase, represent promising candidate biomarkers that could serve as indicators for personalized therapy. Specifically, they might identify patients within this “mast cell-rich” subtype who are more likely to respond to mast cell stabilizers, enabling targeted treatment approaches. Validating these markers could guide the stratification of patients in future clinical trials targeting mast cell pathways.

#### 4.2.3 Emerging Therapies

Emerging therapies represent future directions for disrupting mast cell-mediated pain pathways. GPR30 antagonists represent a promising novel therapeutic strategy for treating endometriosis pain. These antagonists target the GPR30/FGF2/FGFR1 nociceptive signalling axis, inhibiting a key pathway in pain pathogenesis^43^. Preclinical evidence has demonstrated the efficacy of GPR30 antagonists in disrupting pain signalling; notably, the selective antagonist G-36 alleviates endometriosis pain in rat models^74^. Selective GPR30 antagonists have been developed and validated in cancer models. G36 (a GPR30 antagonist) suppressed estrogen-driven tumour growth in endometrial cancer by blocking GPR30-mediated signalling^75^. However, their application in endometriosis pain requires further investigation, including assessment of long-term effects on reproductive and endocrine functions and exploration of synergistic effects with FGFR1 inhibitors for dual-pathway blockade.

NLRP3 antagonists also represent a promising strategy for treating endometriosis pain. Preclinical evidence has demonstrated that NLRP3 inhibitors disrupt the estrogen/mast cell/NLRP3/IL-1β pain axis, thereby targeting a core inflammatory driver 76. Additionally, early human trials support the feasibility of NLRP3/IL-1β blockade in alleviating inflammation-driven pain^44^. Early-phase trials have indicated that NLRP3 antagonists such as DFV890 and selnoflast are not associated with serious adverse events^77^,^78^. However, their application in treating endometriosis pain requires monitoring for off-target effects (such as liver toxicity, as seen with GDC-2394)^79^, and clinical trials in endometriosis patients—with endpoints centred on pain (visual analogue scale [VAS] scores)—are critically needed.

In summary, antagonists targeting GPR30, NLRP3, or other small molecules demonstrate considerable potential for managing endometriosis pain. However, further targeted clinical investigations are imperative to validate their efficacy, safety, and optimal utility in this patient population.

## 5. Conclusion

### 5.1 Summary of Key Findings

**Figure 2:**
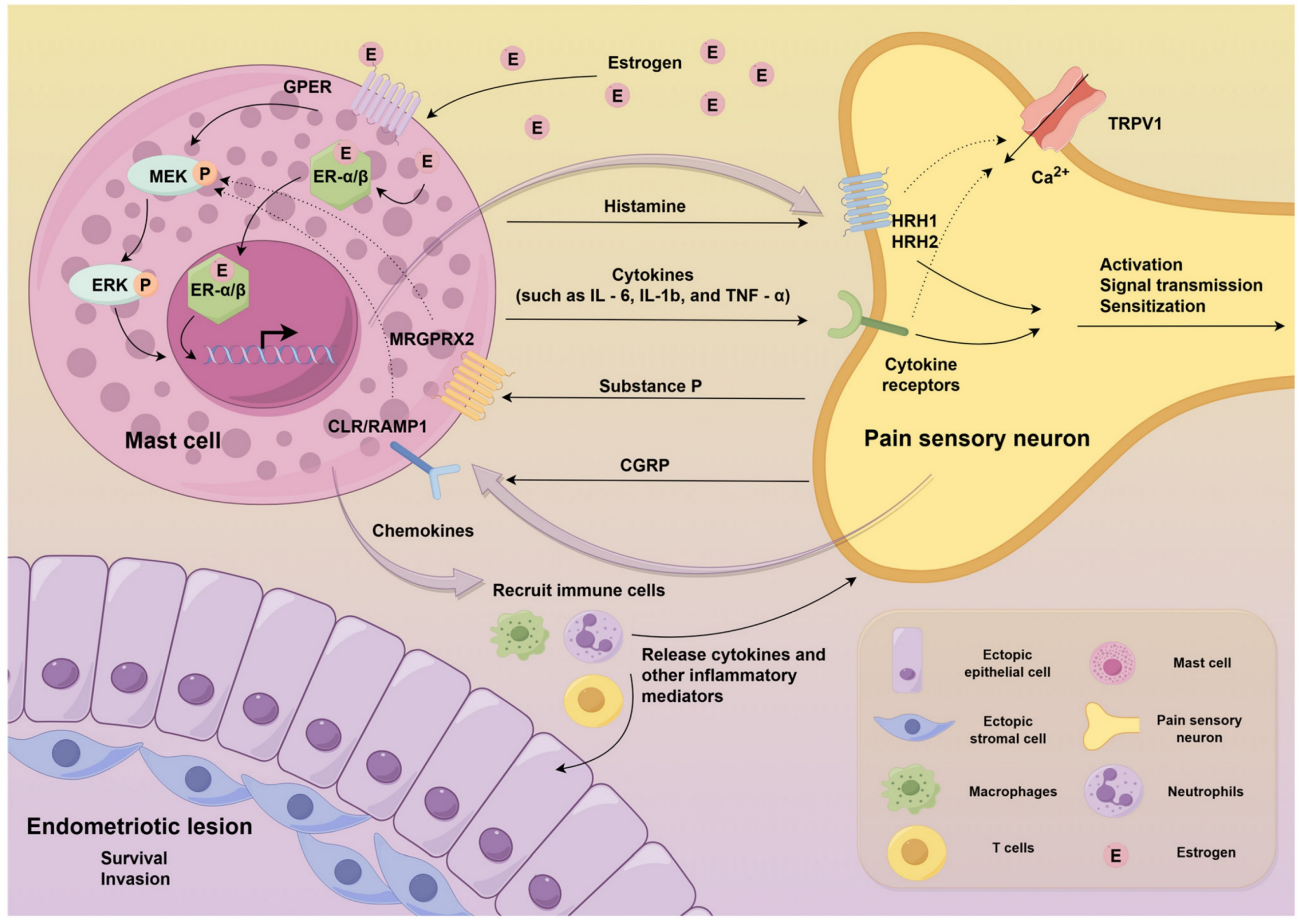
** The molecular mechanisms underlying mast cell-mediated pain sensitization in endometriosis.** Specifically, high concentrations of estrogen in endometriotic lesions mediate the accumulation and activation of mast cells. Upon activation, these mast cells release inflammatory mediators such as histamine, cytokines and chemokines that act on sensory nerve fibres, which enhances pain signalling and contributes to central sensitization. In addition, this complex neuroimmune interaction between mast cells and nerve fibres in endometriotic lesions forms a positive feedback loop that amplifies pain. (Created by Figdraw).

In summary, the role of mast cells in mediating estrogen-induced pain sensitization in endometriosis has been elucidated through a growing body of research. Estrogen, mast cells and nerve fibres form a positive feedback loop that amplifies pain (Figure 2).

Estrogen plays a crucial role in the development and progression of endometriosis. Endometriotic lesions are estrogen dependent, with estrogen promoting the growth, survival, and angiogenesis of these lesions. Moreover, estrogen can directly and indirectly sensitize the nervous system, leading to enhanced pain perception.

Mast cells, key players in the immune and inflammatory responses, are being increasingly recognized for their role in endometriosis-related pain. Mast cells are present in increased numbers and are in an activated state in endometriotic lesions. They express estrogen receptors and their activation and function can be modulated by estrogen. Estrogen-mast cell interactions can lead to increased mast cell accumulation, activation and the release of inflammatory mediators in endometriotic lesions.

The molecular mechanisms underlying mast cell-mediated pain sensitization in endometriosis are complex. Inflammatory mediators released by mast cells, such as histamine, cytokines, and chemokines, play crucial roles. Histamine can directly act on nociceptive neurons, increasing their excitability and promoting pain transmission. Cytokines and chemokines can recruit immune cells, amplify the inflammatory response, and directly sensitize nociceptive neurons. Neuroimmune interactions between mast cells and nerve fibres are also essential. Mast cells and nerve fibres are in close proximity in endometriotic lesions, and they communicate through the release of neurotransmitters and neuropeptides. Substance P and CGRP are important neuropeptides involved in this communication, creating a positive feedback loop that amplifies the pain-signalling pathway. The activation of mast cells in endometriotic lesions can also lead to activation of the central nervous system, resulting in central sensitization and enhanced pain perception.

### 5.2 Clinical Significance and Potential Impact on Patient Care

Research on mast cells in endometriosis has significant clinical implications, particularly in the context of pain management for patients. While both mast cells (MCs) and macrophages contribute to endometriosis-associated inflammation and pain, their temporal responses and mediator profiles highlight distinct roles. Mast cells respond rapidly within minutes of stimulation, releasing presynthesized granules (such as histamine and tryptase) that directly amplify neurogenic inflammation and vascular permeability in endometriotic lesions^23^,^80^. Critically, mast cells exhibit acute sensitivity to estrogen via nongenomic signalling, enabling swift mediator release even at low hormone concentrations^24^. In contrast, macrophages respond more gradually, undergoing polarization shifts that modulate chronic inflammation through cytokine secretion and promote nerve growth in endometriotic lesions^27^,^81^. The broader mediator spectrum of mast cells—spanning neuroactive, fibrogenic, and vasoactive molecules—positions them as early amplifiers of pain signalling. Macrophages primarily sustain chronic inflammation via cytokine cascades. Thus, targeting mast cells offers strategic advantages for early intervention against acute pain, whereas macrophage modulation addresses later-stage tissue remodelling. Additionally, the interaction between mast cells and neurons can lead to neural sensitization and neuroinflammation, contributing to the maintenance of chronic inflammation.

Understanding the role of mast cells in estrogen-induced pain sensitization provides a novel perspective on the pathophysiology of endometriosis-related pain, which can potentially lead to the development of more effective and targeted pain management strategies. One of the key clinical implications is the potential for mast cell-targeted therapies to provide relief for endometriosis patients who do not respond well to current treatments. Mast cell-targeted drugs, such as antihistamines and mast cell stabilizers, offer alternative approaches. Mast cell stabilizers, on the other hand, can prevent mast cell degranulation, thus inhibiting the release of various inflammatory mediators. Translating these findings into clinical practice could provide new treatment options for endometriosis patients, especially those with severe pain that is difficult to control with existing therapies. Moreover, knowledge of mast cell-mediated pain mechanisms can aid in the development of personalized medicine approaches. By analysing the genetic, proteomic, and metabolomic profiles of mast cells from individual patients, it may be possible to identify biomarkers that can predict the response to mast cell-targeted therapies. This would allow clinicians to tailor treatment plans to each patient, choosing the most appropriate drugs, dosages, and treatment durations.

Overall, research on mast cells in endometriosis has the potential to revolutionize the way in which endometriosis-related pain is managed. It offers new treatment options, the possibility of personalized medicine, and a better understanding of disease pathophysiology. Therefore, further research is needed to fully explore the clinical potential of mast cell-targeted therapies and to translate these findings into widespread clinical practice.

## Abbreviations

ERs: Estrogen receptors
GPR30: G-protein-coupled estrogen receptor 30
TRPV1: Transient receptor potential vanilloid 1
IgE: Immunoglobulin E
TLRs: Toll-like receptors
EREs: Estrogen response elements
FGF2: Fibroblast Growth Factor 2
PLC: Phospholipase C
PIP2: Phosphatidylinositol 4,5-bisphosphate
IP3: Inositol trisphosphate
DAG: Diacylglycerol
Ca^2+^: Calcium ions
CGRP: Calcitonin gene-related peptide
IL-6: Interleukin-6
JAK: Janus kinase
STAT: Signal transducer and activator of transcription
TNF-α: Tumour necrosis factor-α
NF-κB: Nuclear factor-κB
MCP-1: Monocyte chemoattractant protein-1
MIP-1α: Macrophage inflammatory protein-1α
NK-1R: Neurokinin-1 receptor
CNS: Central nervous system
